# Changes of the QRS-T angle in Patients Undergoing Transcatheter Aortic Valve Implantation

**DOI:** 10.7150/ijms.130315

**Published:** 2026-03-17

**Authors:** Scharbanu Amirie, Behnam Zadeh, Oliver Bruder, Thomas Schmitz, Karl Toischer, Christoph Julian Jensen

**Affiliations:** 1 Department of Cardiology and Angiology, Contilia Heart and Vascular Center, Elisabeth-Krankenhaus Essen, Essen, Germany.; 2 Ruhr University Bochum, Bochum, Germany.; 3 B. Braun Ambulantes Herzzentrum Kassel, Kassel, Germany.; 4 Department of Cardiology and Pulmonology, University Medical Center Goettingen, Goettingen, Germany.

**Keywords:** QRS-T angle, aortic valve stenosis, transcatheter aortic valve replacement

## Abstract

**Objective:**

This study aimed to investigate changes in the frontal QRST angle in patients who underwent transcatheter aortic valve implantation (TAVI).

**Background:**

The QRS-T angle is a prognostic marker in several clinical settings. The impact of TAVI on the QRS-T angle, especially in the long term, has not been investigated thus far.

**Methods:**

A total of 104 patients undergoing transcatheter aortic valve replacement due to severe aortic stenosis underwent a standardized digital 12-lead ECG for the calculation of the QRS-T angle before and 24 h after the intervention, as well as 30 days after the intervention.

**Results:**

There was a significant and dynamic change in the QRS-T angle during the TAVI procedure. An initial increase in the number of patients with a wider QRS-T angle (>90°) was notable immediately after valve replacement. After 30 days, there was a significant decrease in the number of patients with a wide QRS T-angle.

**Conclusion:**

The QRS-T angle widens early after transcatheter aortic valve replacement and improves below the baseline during a 3 months follow-up. This could be related to the cardiac

ischemic stress during valve intervention. In contrast, QRS-T angle narrowing during follow-up might reflect the normalization of left ventricular hemodynamics and prognostic improvement.

## Introduction

Aortic valve stenosis (AVS) is the most common valvular heart disease in Europe and North America that requires surgical or catheter intervention 1, 2, 3. Prognosis is poor in untreated severe aortic stenosis as soon as symptoms (angina, dyspnea, syncope) develop 4,5,6. Surgical aortic valve replacement (SAVR) has been shown to improve symptoms and survival and restore life expectancy to that of the general population 7,9. However, advanced age and comorbidities leave many patients untreated 8. During the last decade, transcatheter aortic valve implantation (TAVI) has become an established alternative to surgery in elderly patients with high or intermediate surgical risks 10,11.

Individual risk assessment and joint decision-making (heart team approach) are crucial for adequate treatment in patients with AVS. Apart from imaging, a simple derived parameter from routine ECG would be extremely helpful in risk stratification of patients following TAVI. The frontal QRS-T angle, which represents the balance of depolarization to repolarization and, thus, electrical instability, is a promising risk marker 12. The QRS-T angle has a strong prognostic impact in various clinical settings 14-17, and a poor prognosis in patients with a wide QRS-T angle undergoing SAVR has been recently demonstrated 13. In a previous study, we identified a correlation between the extent of myocardial damage caused by ST-elevation myocardial infarction (STEMI), as quantified by late gadolinium enhancement (LGE) on cardiac magnetic resonance imaging (MRI), and a larger QRS-T angle (≥ 90°) 18. In addition, the extent of myocardial fibrosis in patients with hypertrophic cardiomyopathy is associated with a wide QRS-T angle and can therefore be considered a prognostic predictor of mortality 19.

The serial changes in the QRS-T angle and the impact of valve intervention on the QRS-T angle have not yet been investigated.

This study aimed to examine the long-term changes in the frontal QRST-angle in patients undergoing transcatheter aortic valve replacement.

## Methods

In this retrospective trial, patients who underwent transcatheter aortic valve replacement (from January 2014 to December 2014) for symptomatic severe aortic valve stenosis at the Department of Cardiology and Angiology of the Elisabeth Hospital Essen were enrolled. In general, the indications for TAVI followed the current guidelines, and individual decisions were based on the heart team approach for every patient 20. Demographic, imaging, and procedural data were recorded. Routine 12-lead digital ECG was obtained 24 h before, 24 h after TAVI, and at 1-month follow-up. The 12-lead digital ECGs using a Schiller Cardiovit AT 102 plus® were recorded at a speed of 50 mm/s with 10 mm/mV for the limb and precordial leads. The computerized values of the QRS and T-wave axes were automatically provided by the Schiller AT 102 plus® software. The frontal QRS-T angle was calculated as the absolute difference between the frontal QRS- and frontal T-wave axes and expressed as absolute values (Oehler). Due to the retrospective nature of this study informed consent was waived by the ethics committee.

### Statistical analysis

Continuous variables are presented as mean ± standard deviation and were compared using two-way ANOVA. Categorical variables are presented as numbers and percentages and were compared using the chi-square test. Differences between groups were analyzed using a two-way ANOVA. All variables with a univariate *p*-value < 0.05 were included in the final multivariate model. The results of the binary logistic regression model are presented in the same way as the variables in the univariate analysis. All reported *p*-values were two-sided, and a *p*-value <0.05 was considered statistically significant. All statistical analyses were performed using IBM SPSS Statistics version 26.0 (IBM Corp., Armonk, N.Y., USA).

## Results

A total of 104 patients (82.5±6.19 years, 57,69% female) with symptomatic high-degree aortic valve stenosis (mean maximum velocity 4.19±0.68, mean transvalvular gradient 44.21±13.64, mean aortic valve area 0.74±0.20) underwent transcatheter aortic valve replacement. The median ejection fraction was 52.27 ± 11.73. The rate of syncope prior to TAVI was 14%. The mean (± SD) log. EuroSCORE was 22.38±11.29. (Table 1).

a.) QRS T angle pre TAVI:

Patients with a QRS-T angle ≥ 90° (n = 36; mean angle 130.44° ± 28.70°) at baseline experienced episodes of syncope with significantly greater frequency (p = 0.0022) than those with a QRS-T angle < 90° (n = 68). Furthermore, patients with a QRS-T angle < 90° showed a higher rate of pulmonary disease (p = 0.02) and cancer (p = 0.04). (Table 2).

b.) QRS T angle post TAVI

Immediate results post TAVI showed a higher number of patients with a wide QRS-T angle ≥ 90° (n = 48; mean angle 133.81° ± 30.95°) and significantly lower max grades of 64.85 ± 24.03 in comparison to 56 patients with a lower QRS T angle < 90° (max grad 75.47 ± 22.97; p = 0,0256). The left ventricular mass (Devereux) was significantly lower in patients with a QRS-T angle ≥ 90° (219.90 ± 53.01; p = 0.0498). (Table 3).

c.) One-month follow-up data:

The one-month follow-up (30 days) data indicated a decrease in the patient rate with a high QRS-T angle (n = 22) compared to pre-TAVI and a 24-hour analysis post TAVI. The incidence of cancer and pulmonary diseases remained significantly higher in the group of patients with a lower QRS-T angle (< 90°; 0.0097 pulmonary disease; cancer 0.0432). (Table 4).

## Discussion

**Figure 1:**
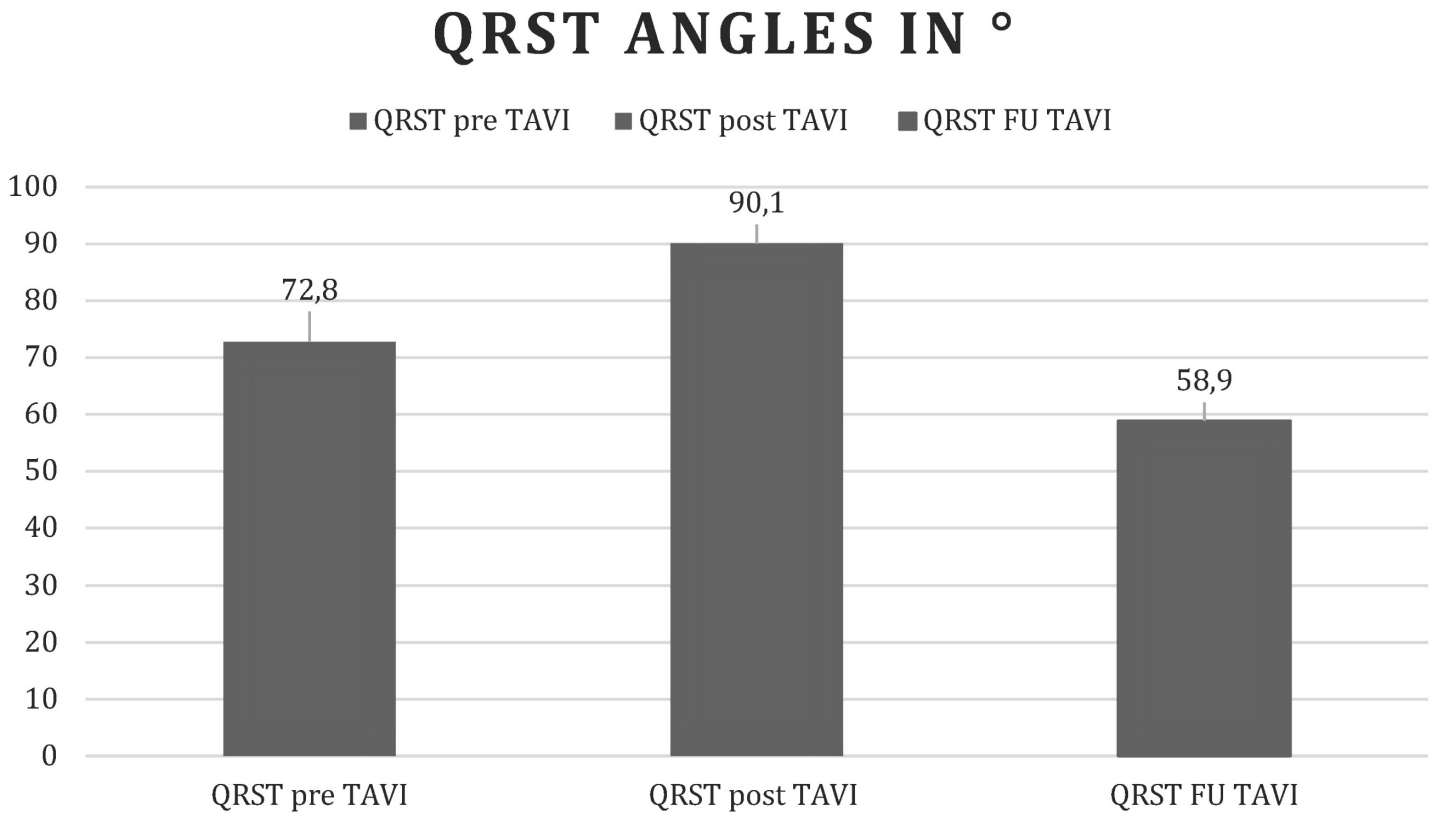
Changes in the QRS-T angle over time. Before, immediately, and 1 month after TAVI. Values are displayed as mean QRST angles in degrees.

Our study shows that the QRS-T angle, an easily accessible risk marker computed with every routine surface ECG, widens early after transcatheter aortic valve replacement, reflecting global ischemic stress during valve intervention, and improves below baseline during follow-up, which in turn reflects the long-term effects of normalization of left ventricular hemodynamics and prognostic improvement (Figure 1).

The QRS-T angle is a marker of electrical instability and poor prognosis. However, evidence of its potential role in patients with aortic valve stenosis is limited. In 34 patients with hemodynamically assessed aortic valve stenosis, the spatial QRS-T angle was correlated with the peak systolic and mean transvalvular gradients. A QRS-T angle above 90°was diagnostic of severe aortic valve stenosis (mean transvalvular gradient of ≥ 50 mmHg) 20. In 372 patients undergoing SAVR, Erturk et al. found that in-hospital and long-term mortality were significantly higher in patients with a wide QRS-T angle above 90° 13. An electrical risk score, including the frontal QRS-T angle, was associated with poor prognosis in patients with severe aortic valve stenosis following TAVI 21.

Only a few studies have focused on changes in the QRS-T angle. Widening of the QRS-T angle during follow-up was independently associated with mortality in 2,929 patients with heart failure 22. In 152 of 455 patients from the Defibrillators in Nonischemic Cardiomyopathy Treatment Evaluation (DEFINITE) trial with serial ECG follow-ups, changes in the QRS-T angle over time were associated with changes in left ventricular function 23. Perez-Alday et al. demonstrated dynamic changes of the prediction of sudden cardiac death by a combination of ECG markers (global electrical heterogeneity index) including the QRS-T angle from 15,716 patients of the Atherosclerosis Risk In Community study 17.

Our study highlights the dynamic changes in the QRS-T angle during the catheter-based treatment of severe aortic stenosis. Changes in the QRS-T angle (narrowing) following successful therapeutic interventions have been demonstrated in patients undergoing thrombolysis for pulmonary artery embolism 25, thrombolysis for ST-segment elevation myocardial infarction 26, percutaneous coronary intervention in patients with chronic total occlusion 27, and improvements in left ventricular function following renal transplantation 28. These findings underscore the sensitivity of the QRS-T angle to alterations in myocardial electrical activity and structural remodeling after intervention.

During TAVI, patients experience extreme hemodynamic fluctuations that impose significant stress on the myocardium and cardiovascular system. These hemodynamic changes trigger a cascade of physiological responses, including myocardial remodeling, characterized by alterations in myocardial fibrosis, cellular architecture, and electrophysiological properties. The left ventricle, which is directly impacted by the relief of valvular obstruction, undergoes substantial electrical remodeling, as reflected in the shifting QRS-T angle values. This remodeling encompasses changes in depolarization and repolarization patterns due to modifications of myocardial conduction pathways and repolarization heterogeneity. Consequently, the QRS-T angle serves not only as a marker of electrical instability but also reflects the underlying structural and functional myocardial recovery. Importantly, the observed QRS-T angle dynamics during and after catheter-based aortic valve implantation suggest that this parameter can be leveraged as a model to investigate the complex interplay between myocardial structural changes, such as fibrosis progression or regression, and electrical remodeling in severe aortic stenosis. Understanding this interplay is crucial because myocardial fibrosis influences both mechanical function and arrhythmogenic potential.

This research has several limitations that need to be recognized. Firstly, the sample size was quite small, which might restrict the applicability of the results to a wider population. Furthermore, the study's retrospective nature limits the ability to determine causal links and could introduce selection bias. The follow-up period was also relatively brief, which hampers the evaluation of long-term changes in QRS-T angle dynamics and their clinical significance. Moreover, the lack of prognostic data, such as patient outcomes related to arrhythmia occurrence or survival, limits the capacity to associate QRS-T angle changes with significant clinical endpoints. These limitations underscore the necessity for prospective studies with longer follow-up durations and thorough outcome monitoring to better understand the prognostic importance of QRS-T angle variations after catheter-based aortic valve implantation.

Given the unique pathophysiological environment created by TAVI, with its abrupt hemodynamic shifts and subsequent myocardial adaptation, this patient population provides a valuable opportunity to study disease progression and recovery using noninvasive ECG markers, such as the QRS-T angle. Future research focusing on correlating QRS-T angle changes with advanced imaging modalities that assess myocardial fibrosis and function could yield deeper insights into the mechanisms of electrical and structural remodeling.

Moreover, integrating cutting-edge computational approaches, such as deep learning algorithms applied to large-scale patient data, could enhance the predictive accuracy and clinical utility of QRS-T angle monitoring 29. Such advancements may facilitate personalized risk stratification and optimize post-TAVI management by identifying patients at a higher risk for adverse remodeling or arrhythmic events.

In summary, the QRS-T angle emerges as a promising, easily accessible biomarker that reflects the dynamic myocardial and electrical remodeling processes occurring during and after catheter-based aortic valve replacement, offering significant potential for both clinical monitoring and mechanistic research in patients with severe aortic stenosis.

## AI Usage Statement

Generative AI was used in the creation of the graphical abstract figure.

## Figures and Tables

**Table 1:** Demographic, imaging, angiographic and QRS-T angle data at baseline

	n = 104
**Gender w (%)**	57.7
**Age (years)**	82.5 + 6.2
**BMI (kg/m²)**	26.8 + 4.8
**CAD (%)**	52.9
**1 vessel**	11.5
**2 vessels**	7.7
**3 vessels**	33.7
**In %**	
**prev MI**	5.8
**prev CABG**	22.1
**prev PCI**	26.9
**In %**	
**Diabetes**	31.7
**Hypertension**	98.1
**Hypercholesterolemia**	68.3
**Smoker**	6.7
**Family history**	5.8
**Ckd**	28.9
**Carotid disease**	14.4
**Periph. Disease**	18.3
**Af**	9.6
**Pulmonary disease**	24.1
**Cancer**	9.6
**Log Euroscore-I in %**	22.4 + 11.3
**Syncope in %**	14.4
**CCS (I-IV)**	1.1 + 0.9
**NYHA (I-IV)**	3.0 + 0.6
**QRS-T angle in degree °**	
**pre TAVI**	72.8 + 49.5
**post TAVI**	90.1 + 49.6
**FU TAVI**	58.9 + 47.4
**Lab results**	
**Creatinine (mg/dl)**	1.2 + 0.7
**Hb (g/dl)**	12.0 + 1.6
**TnT (ng/l)**	0.1 + 0.1
**Creatinine post (mg/dl)**	1.3 + 0.8
**Hb post (g/dl)**	9.8 + 1.5
**TnT max post (ng/l)**	0.2 + 0.2
**EF pre in %**	52.2 + 11.7
**Left atrial diameter (mm)**	43.3 + 6.0
**Left ventricular enddiastolic diameter (mm)**	48.1 + 8.0
**Max V (m/s)**	4.2 + 0.7
**Max grad (mmHg)**	71.0 + 24.0
**Pmean pre (mmHg)**	44.2 + 13.6
**AVA pre (cm²)**	0.7 + 0.20
**EF post in %**	53.0 + 9.4
**Velocity (m/s)**	2.2 + 0.6
**Pmean post (mmHg)**	12.3 + 5.3
**Pmax post (mmHg)**	23.3 + 9.1
**Valve dimensions (mm)**	26.5 + 2.1
**Access transfemoral (%)**	99.0
**Balloon size in mm**	22.1 + 4.8
**P-P Gradient pre (mmHg)**	45.5 + 19.6
**P-P Gradient post (mmHg)**	6.2 + 5.2

**Table 2:** QSR-T angle and clinical data at baseline stratified by QRS-T angle.

	QRS-T ≥ 90 pre TAVI	QRST < 90 pre TAVI	p value
	(n = 36)	(n = 68)	
**Gender w (%)**	64.0	55.9	0.28
**Age (years)**	83.2 ± 5.4	82.4 ± 6,5	0.22
**BMI (kg/m²)**	27.1 ± 4.12	26.5 ± 5.0	0.25
**CAD (%)**	47.22	55.9	0.20
**1 vessel**	5.6	14.7	
**2 vessels**	11.1	5.9	
**3 vessels**	30.6	35.3	
**In %**			
**prev MI**	8.3	4.4	0.21
**prev CABG**	19.4	23.5	0.32
**prev PCI**	22.2	27.9	0.27
**In %**			
**Diabetes**	41.7	26.5	0.13
**Hypertension**	100.0	97.1	0.15
**Hypercholesterolemia**	77.8	63.2	0.06
**Smoker**	2.8	8.8	0.12
**Family history**	5.6	5.9	0.47
**Ckd**	27.8	27.9	0.49
**Carotid disease**	11.1	16.1	0.24
**Periph disease**	16.7	20.6	0.31
**Af**	5.6	10.3	0.13
**Pulmonary disease**	11.1	29.4	**0.0269**
**Cancer**	2.8	13.2	
**Log Euroscore in %**	22.5 ± 13.6	22.4 ± 9.8	0.48
**Syncope in %**	27.8	7.4	**0.0022**
**CCS (I-IV)**	1.3 ± 0.8	1.0 ± 1.0	0.16
**NYHA (I-IV)**	3.0 ± 0.7	2.9 ± 0.6	0.50
**QRS-T angle** °	130.4 ± 28.7	41.7 ± 23.8	**< 0.001**
**Crea base (mg/dl)**	1.2 ± 0.7	1.2 ± 0.8	0.50
**Hb base (g/dl)**	12.0 ± 1.4	12.1 ± 1.7	0.36
**TnT base (ng/l)**	0.02 ± 0.02	0.1 ± 0.2	0.38
**crea post (mg/dl)**	1.3 ± 1.0	1.2 ± 0.8	0.28
**Hb post (g/dl)**	9.7 ± 1.3	9.9 ± 1.6	0.31
**TnT max post (ng/l)**	0.3 ± 0.2	0.2 ± 0.1	**0.0178**
**EF base in %**	51.4 ± 11.6	52.4 ± 9.9	0.38
**Left atrial diameter (mm)**	42.4 ± 5.4	42.8 ± 6.2	0.28
**Left enddiastolic diameter (mm)**	43.1 ± 5.8	47.6 ± 7.8	0.41
**Max V (m/s)**	4.3 ± 0.6	4.2 ± 0.7	0.32
**Max grad (mmHg)**	71.1 ± 25.8	71.4 ± 23.0	0.48
**Pmean mmHg base (mmHg)**	45.1 ± 10.7	43.9 ± 15.0	0.35
**AVA base (cm²)**	0.8 ± 0.2	0.7 ± 0.2	0.32
**EF post (%)**	52.4 ± 9.9	52.4 ± 9.1	0.45
**Velocity (m/s)**	2.3 ± 0.5	2.3 ± 0.5	0.19
**Pmean post (mmHg)**	10.9 ± 5.1	13.2 ± 4.9	0.15
**Pmax post (mmHg)**	20.9 ± 9.9	23.7 ± 8.5	0.16
**Valve dimensions (mm)**	26.9 ± 1.9	25.4 ± 2.0	0.0531
**Access in %**	100	100	
**Balloon size (mm)**	22.4 ± 1.6	21.8 ± 6.0	0.44
**P-P Gradient pre (mmHg)**	44.5 ± 14.5	44.5 ± 21.6	0.34
**P-P Gradient post (mmHg)**	7.5 ± 4.1	5.6 ± 5.4	0.14

**Table 3:** QSR-T angle and clinical data post-intervention stratified by QRS-T angle.

	QRS-T ≥ 90 post TAVI	QRST < 90 post TAVI	p value
	(n = 48)	(n = 56)	
**Gender w (%)**	60.4	54.4	0.30
**Age (years)**	83.3 ± 6.2	81.8 ± 6.1	0.11
**BMI (kg/m²)**	26.8 ± 4.7	26.5 ± 5.0	0.47
**CAD (%)**	49.1	39.7	0.26
**1 vessel**	5.9	11.8	
**2 vessels**	7.4	4.4	
**3 vessels**	26.5	25.0	
**In %**			
**prev MI**	8.3	3.5	0.15
**prev CABG**	25.0	19.3	0.26
**prev PCI**	27.9	29.8	0.20
**In %**			
**Diabetes**	27.1	35.1	0.31
**Hypertension**	97.9	96.5	0.46
**Hypercholesterolemia**	77.1	59.7	0.04
**Smoker**	6.3	7.1	0.42
**Family history**	2.1	8.8	0.07
**Ckd**	31.3	26.3	0.31
**Carotid disease**	18.8	10.5	0.12
**Periph disease**	14.6	21.1	0.19
**Af**	10.4	8.8	0.30
**Pulmonary disease**	12.5	33.3	0.08
**Cancer**	8.3	10.5	0.34
**Log euroscore**	21.8 ± 11.6	22.9 ± 11.0	0.33
**Syncope in %**	18.8	10.5	0.12
**Ccs (i-iv)**	1.2 ± 1.0	1.1 ± 0.9	0.22
**Nyha (i-iv)**	2.9 ± 0.7	3.1 ± 0.6	0.11
**QRS-T angle °**	133.8 ± 31.0	52.5 ± 26.3	
**Lab results**			
**Creatinine pre (mg/dl)**	1.3 ± 0.7	1.2 ± 0.8	0.39
**Hb pre (g/dl)**	12.1 ± 1.5	12.0 ± 1.7	0.08
**Tnt pre (ng/l)**	0.1 ± 1.6	0.04 ± 0.02	0.18
**Creatinine post (mg/dl)**	1.2 ± 1.5	1.2 ± 0.9	0.39
**Hb post (g/dl)**	9.9 ± 1.5	9.8 ± 1.5	0.41
**Tnt max post (ng/l)**	0.2 ± 0.2	0.2 ± 0.1	0.08
**EF pre in %**	52.6 ± 11.6	51.7 ± 11.8	0.39
**Left atrial diameter (mm)**	43.0 ± 5.1	43.6 ± 6.7	0.32
**Left ventricular enddiastolic diameter (mm)**	47.6 ± 8.6	48.5 ± 7.5	0.29
**Max V (m/s)**	4.1 ± 0.6	4.3 ± 0.7	0.06
**Max grad (mmhg)**	64.9 ± 24.0	75.5 ± 23.0	**0.0256**
**Pmean mmhg pre (mmhg)**	43.2 ± 11.5	44.9 ± 15.2	0.29
**AVA pre (cm²)**	0.7 ± 0.2	0.8 ± 0.2	0.32
**EF post (%)**	54.7 ± 8.9	51.9 ± 9.5	0.15
**Velocity (m/s)**	2.2 ± 0.6	2.2 ± 0.5	0.49
**Pmean post (mmhg)**	11.8 ± 5.1	12.5 ± 5.3	0.34
**Pmax post (mmhg)**	22.1 ± 10.6	24.0 ± 7.8	0.25
**Valve dimensions (mm)**	26.5 ± 1.9	26.4 ± 2.2	0.41
**Access TF in %**	100	100	
**Balloon size (mm)**	20.2 ± 7.2	23.1 ± 1.8	**0.0182**
**P-P Gradient pre (mmhg)**	46.4 ± 15.8	45.1 ± 21.6	0.40
**P-P Gradient post (mmHg)**	6.0 ± 4.7	6.3 ± 5.5	0.42

**Table 4:** QSR-T angle and clinical data at follow-up stratified by QRS-T angle.

	QRS-T ≥ 90 FU TAVI	QRST < 90 FU TAVI	p value
	(n = 22)	(n = 82)	
**Gender w (%)**	45.46	61.0	0.10
**Age (years)**	83.0 ± 5.8	82.4 ± 6.23	0.34
**BMI (kg/m²)**	26.3 ± 4.0	26.9 ± 5.0	0.27
**CAD in %**	66.6	50.0	0.13
**1 vessel**	2.9	14.7	
**2 vessels**	2.9	8.8	
**3 vessels**	14.7	36.8	
**In %**			
**Prev MI**	9.1	4.9	0.23
**Prev CABG**	27.3	20.7	0.26
**Prev PCI**	40.9	29.3	0.15
**In %**			
**Diabetes**	40.9	29.3	0.41
**Hypertension**	100	97.6	0.23
**Hypercholesterolemia**	77.3	65.9	0.16
**Smoker**	13.6	4.9	0.07
**Family history**	0	7.3	0.10
**Ckd**	13.6	32.9	0.04
**Carotid disease**	4.6	17.1	0.07
**Periph disease**	18.2	18.3	0.50
**Af**	0	12.2	0.40
**Pulmonary disease**	9.1	28.1	**0.0097**
**Cancer**	0	12.2	**0.0432**
**Log euroscore (%)**	21.1 ± 12.4	22.7 ± 11.0	0.28
**Syncope in %**	13.6	14.6	0.45
**Ccs (i-iv)**	1.1 ± 0.8	1.2 ± 0.9	0.46
**Nyha (i-iv)**	3.1 ± 0.2	3.0 ± 0.7	0.30
**QRS-T angle °**	133.2 ± 27.4	38.7 ± 27.4	**< 0.001**
**Lab results**			
**Creatinine pre (mg/dl)**	1.1 ± 0.3	1.3 ± 0.8	0.16
**Hb pre (g/dl)**	12.5 ± 1.3	11.9 ± 1.7	0.10
**Tnt pre (ng/l)**	0.03 ± 0.01	0.1 ± 0.1	0.27
**Crea post (mg/dl)**	1.1 ± 0.4	58.5 ± 31.5	0.15
**Hb post (g/dl)**	10.1 ± 1.8	9.8 ± 1.4	0.29
**Tnt max post (ng/l)**	0.2 ± 0.2	0.2 ± 0.2	0.39
**EF pre in %**	53.0 ± 11.3	52.1 ± 11.9	0.38
**Left atrial diameter (mm)**	43.3 ± 7.4	43.3 ± 5.5	0.50
**Left ventricular enddiastolic diameter (mm)**	47.7 ± 7.0	48.2 ± 8.3	0.40
**Max V (m/s)**	4.2 ± 0.7	4.0 ± 0.7	0.48
**Max grad (mmhg)**	71.6 ± 21.2	70.8 ± 24.7	0.45
**Pmean pre (mmhg)**	44.0 ± 9.0	44.3 ± 14.5	0.47
**AVA pre (cm²)**	0.8 ± 0.2	0.7 ± 0.2	0.48
**EF post (%)**	53.0 ± 7.2	52.9 ± 9.7	0.49
**Velocity (m/s)**	2.2 ± 0.6	2.2 ± 0.6	0.46
**Pmean post (mmhg)**	13.6 ± 5.9	12.0 ± 5.1	0.22
**Pmax post (mmhg)**	24.2 ± 7.6	23.1 ± 9.4	0.38
**Valve dimensions (mm)**	27.1 ± 2.2	22.0 ± 5.1	**0.0404**
**Access TF in %**	100	100	
**Balloon size (mm)**	22.7 ± 1.5	22.0 ± 5.1	0.37
**P-P Gradient Pre (mmHg)**	43.1 ± 16.6	46.0 ± 20.1	0.35
**P-P Gradient Post (mmHg)**	7.6 ± 6.9	6.0 ± 4.8	0.21
